# *Leishmania* Hijacks Myeloid Cells for Immune Escape

**DOI:** 10.3389/fmicb.2018.00883

**Published:** 2018-05-07

**Authors:** María Martínez-López, Manuel Soto, Salvador Iborra, David Sancho

**Affiliations:** ^1^Immunobiology Laboratory, Fundación Centro Nacional de Investigaciones Cardiovasculares “Carlos III”, Madrid, Spain; ^2^Departamento de Biología Molecular, Centro de Biología Molecular Severo Ochoa – Consejo Superior de Investigaciones Científicas–Universidad Autónoma de Madrid, Madrid, Spain; ^3^Department of Immunology, Complutense University School of Medicine and 12 de Octubre Health Research Institute (imas12), Madrid, Spain

**Keywords:** myeloid cell, *Leishmania*, immune escape, neutrophils, macrophages, dendritic cells

## Abstract

Protozoan parasites of the *Leishmania* genus are the causative agents of leishmaniasis, a group of neglected tropical diseases whose clinical manifestations vary depending on the infectious *Leishmania* species but also on host factors. Recognition of the parasite by host myeloid immune cells is a key to trigger an effective *Leishmania*-specific immunity. However, the parasite is able to persist in host myeloid cells by evading, delaying and manipulating host immunity in order to escape host resistance and ensure its transmission. Neutrophils are first in infiltrating infection sites and could act either favoring or protecting against infection, depending on factors such as the genetic background of the host or the parasite species. Macrophages are the main host cells where the parasites grow and divide. However, macrophages are also the main effector population involved in parasite clearance. Parasite elimination by macrophages requires the priming and development of an effector Th1 adaptive immunity driven by specific subtypes of dendritic cells. Herein, we will provide a comprehensive outline of how myeloid cells regulate innate and adaptive immunity against *Leishmania*, and the mechanisms used by the parasites to promote their evasion and sabotage. Understanding the interactions between *Leishmania* and the host myeloid cells may lead to the development of new therapeutic approaches and improved vaccination to leishmaniases, an important worldwide health problem in which current therapeutic or preventive approaches are limited.

## Introduction

The trypanosomatid protozoa *Leishmania* spp. belonging to the order kinetoplastida are the causative agents of leishmaniases, whose clinical manifestations can range from cutaneous, mucocutaneous or diffuse cutaneous forms to visceral forms, depending on both the parasite species and the host’s immune response (Pace, 2014). *Leishmania* is a digenetic parasite, whose life cycle involves two hosts, the insect vector and a vertebrate host. *Leishmania* parasites are transmitted to the vertebrate host by the bite of infected female sandflies belonging to the genera Phlebotomus and Lutzomyia (Akhoundi et al., 2016). Inside the sandflies the extracellular flagellated, motile form, called procyclic promastigotes generate the infective, non-dividing metacyclic promastigotes, which are inoculated into the host during blood feeding. Once there, *Leishmania* become into the aflagellate intracellular form, called amastigotes, that undergo replication within host cells, mainly phagocytes such as macrophages. The transmission cycle is complete when infected phagocytes are taken up during a sandfly blood meal, and amastigotes then convert into promastigotes in the sandfly midgut. As a successful parasite, *Leishmania* has developed strategies to evade host immune mechanisms in order to survive within the host. The ability of *Leishmania* to maintain a chronic infectious state within its host depends largely on its immune evasion potential (Geiger et al., 2016). We will review how myeloid cells drive innate and adaptive immunity against *Leishmania* and how the parasites escape host resistance.

## Innate and Adaptive Immunity Against *Leishmania*

The generation of protective immunity against *Leishmania* requires the cooperation between the innate and adaptive host immune cells. Clearance of *Leishmania* parasites that promotes healing requires IFN-γ-producing effector cells, mainly CD4^+^ T helper 1 (Th1). IFN-γ production by NK cells (Bajenoff et al., 2006) and type 1 CD8^+^ T cells (Belkaid et al., 2002b) also correlates with protection against *L. major* in mice, whereas CD8^+^ T cells play an important role in controlling visceral leishmaniasis (Stäger and Rafati, 2012). However, cytotoxic T lymphocytes (CTLs) play a detrimental role during infection with other *Leishmania* species, such as *L. braziliensis* (Novais and Scott, 2015). IFN-γ signaling in infected macrophages promotes expression of inducible nitric oxide (NO) synthase (iNOS, NOS2) and NO production that, together with reactive oxygen species (ROS) generated during phagocytosis, are essential to kill intracellular parasites (Bogdan et al., 1990; Green et al., 1990). However, *L. amazonensis* are resistant to IFN-γ-mediated killing, and parasite control during the early stages of infection in mice is independent of this cytokine (Kima and Soong, 2013). Besides IFN-γ, other inflammatory cytokines, such as TNF, can activate the infected macrophages in an autocrine manner to produce NO (Bronte and Zanovello, 2005). On the contrary, CD4^+^ T helper 2 (Th2)-related cytokines, such as IL-4, IL-13, IL-10, and antibody production are associated with alternative activated macrophages (Gordon, 2003), which favors parasite survival inside the macrophages (Kropf et al., 2005), and a non-healing phenotype (Scott et al., 1988; Heinzel et al., 1989; Chatelain et al., 1992; Sacks and Noben-Trauth, 2002).

Although macrophages are the primary host cell for *Leishmania* parasites, monocytes, dendritic cells (DCs) and neutrophils can be infected and contribute differentially to the immune response and the outcome of the infection. Acting as a bridge between innate and adaptive immune system, DCs have a prominent role for the development of immune response against the parasite. *Leishmania* infection of DCs results in IL-12 production (Marovich et al., 2000), an essential cytokine for the polarization of naïve T cells toward Th1 subset and subsequent IFN-γ production to control the infection (Heinzel et al., 1993; Sypek et al., 1993; von Stebut et al., 1998). DCs derived from inflammatory monocytes (moDCs) and the migratory CD103^+^ DCs are the main source of IL-12 upon *Leishmania* infection (Leon et al., 2007; Martinez-Lopez et al., 2015).

*Leishmania* infection resolution generates a long-lasting immunity to reinfection mediated primarily by a population of short-lived *Leishmania*-specific effector CD4^+^ T cells maintained by low number of parasites that persist after resolution (Peters et al., 2014). Apart from this, *Leishmania*-specific effector memory T (TEM) cells and central memory T (TCM) cells are detected upon *Leishmania* infection (Zaph et al., 2004; Colpitts et al., 2009). Only TCM cells can proliferate, differentiate into effector T cells, and migrate to the lesion site, protecting the host against the infection (Zaph et al., 2004). In addition, CD4^+^ T resident memory cells (TRM) have been identified at sites distant from the primary lesion in *L. major* immune mice and increase the ability of circulating effector cells to mediate protection against the infection (Glennie et al., 2015). After resolution of infection, there are also CD8^+^ T cells, which can contribute to host protection after reinfection or vaccination (Gurunathan et al., 1997; Muller et al., 1997; Rhee et al., 2002; Colmenares et al., 2003; Jayakumar et al., 2011). Understanding how parasites subvert the host innate immune response could help to target these mechanisms in future vaccine strategies that promote more effective and longer-term protection in leishmaniasis mediated by these TCM and TRM cells.

## *Leishmania* Targeting Neutrophils as First Line of Defense

Neutrophils are recruited early after *Leishmania* infection in response to several factors derived from the host, the sand fly, or the parasite itself. These cells contribute to kill the invading pathogens by formation of neutrophil extracellular traps (NETs) or by a potent oxidative burst generation and granule-derived toxic compound secretion in the surrounding environment or into the phagosome (Kolaczkowska and Kubes, 2013). Neutrophils from visceral leishmaniasis patients are highly activated and degranulated (Yizengaw et al., 2016). In addition, CCL3 secreted by neutrophils also attracts monocytes to the site of infection in an experimental model of *L. major* infection (Charmoy et al., 2010b). However, depending on the parasite species and the host, neutrophils can contribute to parasite elimination, or, conversely, favor immune escape by the parasite (Carlsen et al., 2015; de Menezes et al., 2016; Hurrell et al., 2016). Notably, the parasite itself can cause a delay in neutrophil apoptosis that allows parasite replication within these cells (see Regli et al., 2017 for a recent review on how *Leishmania* parasites are able to survive into these myeloid cells).

### NETs in *Leishmania* Infection

**FIGURE 1:**
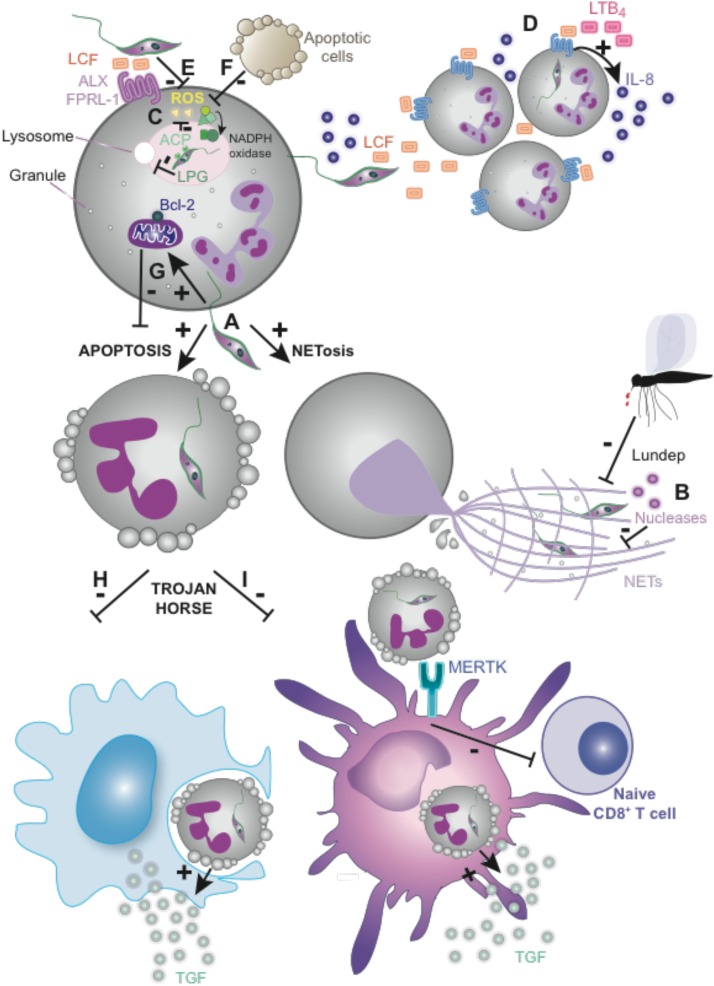
Some *Leishmania* species evade neutrophils and target them for silent transmission. **(A)** *L. major* and *L. amazonensis* promastigotes induce apoptosis and/or NETosis in neutrophils. **(B)** NET-mediated killing inhibition by *L. infantum* and *L. major* nucleases and vector’s endonuclease (Lundep). **(C)** LPG from *L. donovani* or *L. major* contributes to evade phagosome maturation while ACP and LCF from *L. donovani* inhibit the respiratory burst. **(D)** Recruitment of neutrophils to the site of the infection is mediated by LTB4 and might be induced by LCF from several *Leishmania* species, including *L. major*, *L. donovani*, and *L. aethiopica*. **(E)** LCF from the mentioned species inhibits the oxidative burst via the LXA_4_ receptor (ALX/FPRL-1) *in vitro*. **(F)** Apoptotic cell-mediated ROS downregulation promoting *L. major* survival. **(G)** *L. major* extends neutrophils lifespan by induction of anti-apoptotic protein Bcl-2 within other mechanisms (see the main text for more detailed information). **(H,I)** Silent transmission of *Leishmania* or “Trojan Horse” hypothesis. **(H)** Phagocytosis of apoptotic neutrophils containing intact parasites promotes an anti-inflammatory response by macrophages. **(I)** The engagement of MERTK on DCs by infected apoptotic neutrophils, upon infection with *L. major* Friedlin V1 strain, inhibits CD8^+^ T-cell priming. LPG, lipophosphoglycan; ACP, tartrate-resistant acid phosphatase; LCF, *Leishmania* chemotactic factor; LTB_4_, leukotriene B4; LXA_4_, lipoxin A4; ROS, reactive oxygen species; Bcl-2, B-cell lymphoma 2; MERTK, receptor tyrosine kinase Mer.

Neutrophils release NETs composed by granule proteins together with chromatin that form extracellular fibers that can kill microorganisms (Brinkmann et al., 2004). This process can be ROS-dependent and concludes with the death of the neutrophil by a process named NETosis (Fuchs et al., 2007; Kirchner et al., 2012). In addition, there is a ROS-independent early NETosis (Pilsczek et al., 2010). *Leishmania* promastigotes induce both types of NET formation in human and mouse neutrophils both *in vitro* and *in vivo* (**Figure 1A**) (Rochael et al., 2015; Regli et al., 2017). Depending on the species, some promastigotes are resistant to NET-mediated killing as described for *L. mexicana* in mice and *L. donovani* in humans, while others are susceptible as demonstrated for *L. amazonensis* in response to human neutrophils, and this is dependent on lipophosphoglycan (LPG), a glycolipid molecule abundantly found in the surface of the promastigote forms (Guimaraes-Costa et al., 2009; Gabriel et al., 2010; Hurrell et al., 2015). Moreover, *Leishmania* can escape from NET-mediated killing, through the expression of nucleases, as well as by the presence of endonuclease (Lundep) in the vector’s saliva, allowing parasites to survive (**Figure 1B**), as occurred in the interaction between *L. infantum* and *L. major* and human neutrophils (Chagas et al., 2014; Guimaraes-Costa et al., 2014). On the other hand, NETs formation in response to *Leishmania* can interfere with the generation of adaptive immunity, given that NETs isolated from human neutrophils activated by *L. amazonensis* promastigotes are able to inhibit monocyte-derived DCs differentiation and function, thus favoring parasite survival (Barrientos et al., 2014; Guimaraes-Costa et al., 2017).

### Inhibition of Neutrophil-Mediated Oxidative Burst

*Leishmania* promastigote has evolved to survive within neutrophils following phagocytosis. *Promastigotes* from some *Leishmania* species like *L. donovani* or *L. major* express particular molecules, like LPG, that inhibit phagosome maturation (Gueirard et al., 2008; Mollinedo et al., 2010). In addition, others molecules from *L. donovani*, like tartrate-resistant acid phosphatase (ACP) and *Leishmania* chemotactic factor (LCF) inhibit the respiratory burst, (**Figure 1C**) (Remaley et al., 1984; Al Tuwaijri et al., 1990; Wenzel and Van Zandbergen, 2009). LCF from some *Leishmania* species, including *L. major*, *L. aethiopica*, and *L. donovani*, shares features of both LTB_4_ and LXA_4_ mediators. Similar to LTB_4_, LCF contributes to increased neutrophil recruitment *in vitro* either directly or by inducing IL-8 secretion by neutrophils (**Figure 1D**). Similar to LXA_4_, LCF from the cited species, increases parasite engulfment and survival within neutrophils *in vitro* through the inhibition of oxidative burst, mediating its effects via the LXA_4_ receptor (ALX/FPRL-1) (**Figure 1E**) (van Zandbergen et al., 2002). In line with this, *L. major*-infected neutrophils release an increased amount of LTB_4_, whereas LXA_4_ production is reduced, contributing to the initial establishment of the infection (Plagge and Laskay, 2017). On the other hand, the presence of apoptotic cells at the site of infection could contribute to the parasite evasion of the oxidative-mediated killing, since apoptotic cells promote *L. major* survival within neutrophils by downregulating ROS production (**Figure 1F**) (Salei et al., 2017).

### Extending Neutrophil Lifespan

Neutrophils could be considered non-suitable host cells for intracellular parasites due to their short lifespan. Notwithstanding, *Leishmania* infection increases the survival of neutrophils both *in vitro* and *in vivo* (Aga et al., 2002). *L. major* increases neutrophil lifespan by activation of ERK1/2 and induction of anti-apoptotic proteins Bcl-2 and Bfl-1 (**Figure 1G**) (Sarkar et al., 2013). However, the neutrophil response to *Leishmania* infection may depend on its location, since *L. major* delays mouse peritoneal and human blood-derived neutrophil apoptosis (Aga et al., 2002; Charmoy et al., 2010a), but this apoptosis delay is not observed in infected mouse dermal neutrophils (Ribeiro-Gomes et al., 2012).

## Macrophages as Key Hosts and Effectors Against *Leishmania*

*Leishmania* infects macrophages directly after being released from neutrophils (Peters et al., 2008) or following the phagocytosis of apoptotic neutrophils containing intact parasites. The later mechanism mediates a silent transmission of *Leishmania* promastigotes to macrophages and couples to the triggering of an anti-inflammatory response associated to uptake of apoptotic cells, with TGF-β secretion, which favors the survival and division of parasites within the macrophages in a model of transmission so that called “Trojan Horse” (**Figure 1H**) (Laskay et al., 2003, 2008; John and Hunter, 2008). MIP-1β secretion by infected neutrophils favors attraction of macrophages to the site of the infection (van Zandbergen et al., 2004). This process requires *Leishmania*-mediated neutrophil apoptosis, which does not occur in all species (Hurrell et al., 2015). In addition, *in vivo* imaging revealed that parasites can also escape dying neutrophils to infect macrophages, which was termed the ‘Trojan rabbit’ strategy (Ritter et al., 2009). Once inside macrophages, *Leishmania* amastigotes differentiate and multiply, which requires manipulating macrophage function to escape ROS generation and the action of lysosomal enzymes and the acidic milieu of the phagolysosome. In addition, parasites modulate the cytokine repertoire secreted by the infected macrophages and their ability to act as antigen presenting cells, in order to avoid a proper generation of the adaptive immune response (de Menezes et al., 2016).

### Interfering Phagosome Maturation in Macrophages

**FIGURE 2:**
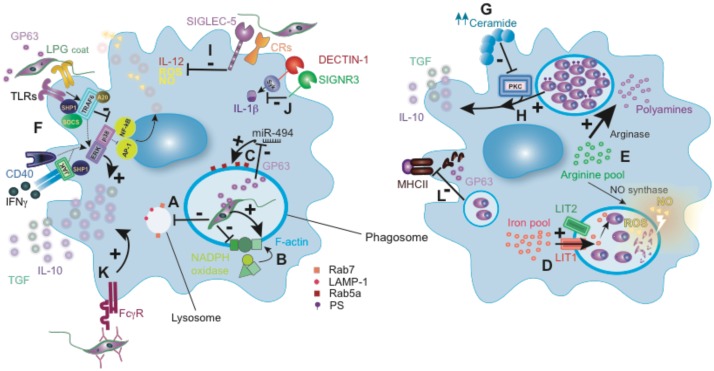
*Leishmania* subversion of macrophage-mediated killing. **(A)** Delayed phagosome maturation by affecting the expression of late endosomal markers LAMP-1 and Rab7, as occurred with *L. donovani* **(B)** LPG from *L. donovani* mediates F-actin accumulation and LPG from *L. donovani* or *L. major* prevents NADPH oxidase complex assembly in the phagosome. **(C)** *L. donovani* metalloprotease GP63 modulation of phagosome Rab5a expression. **(D)** *L.* amazonensis-mediated host iron pool manipulation. **(E)** Some *Leishmania* species (detailed in the main text) mediate arginase upregulation to favor polyamines synthesis and parasite survival. **(F)** JAK/MAPK signaling inhibition by SOCs, SHP1 and A20 described for some *Leishmania* species, including *L. major*, *L. donovani*, *L. mexicana* and *L. amazonensis*. **(G)** PKC inhibition by *L. donovani* amastigotes-mediated ceramide overexpression. **(H)** PS expression by *L. amazonensis* amastigotes favors the production of anti-inflammatory cytokines. **(I)** *L. donovani* uses Siglec-5 and *L. major* some CRs to silence the macrophages pro-inflammatory response. **(J)** *L. infantum* SIGNR3 interaction inhibits Dectin-1 mediated IL-1β production. **(K)** Opsonized-*L. major* parasites recognition by FcγR promotes anti-inflammatory cytokines. **(L)** MHC-II degradation by GP63 from *L. amazonensis* and *L. donovani* amastigotes. Rab7, Ras-related protein 7; LAMP-1, lysosomal-associated membrane protein 1; LPG, lipophosphoglycan; Rab5a, Ras-related protein 5A; GP63, glycoprotein 63; LIT1, *Leishmania* iron transporter 1; LIT2, *Leishmania* iron transporter 2; ROS, reactive oxygen species; NO, nitric oxide; TLRs, Toll-like receptors; SHP1, Src homology region 2 domain-containing phosphatase-1; SOCS, suppressors of cytokine signaling; A20, tumor necrosis factor alpha-induced protein 3; PS, phosphatidylserine; CRs, complement receptors; FcγR, Fcγ receptor.

*Leishmania* parasites delay phagosome formation and maturation, as shown by hindered expression of late endosomal markers LAMP-1 and Rab7, as occurred with *L. donovani* (**Figure 2A**) (Scianimanico et al., 1999; Seguin and Descoteaux, 2016). In addition, some *Leishmania* (such as *L. amazonensis* and *L. mexicana*) promote the formation of large parasitophorous vacuoles by the lysosomal trafficking protein to dilute the leishmanicidal effect of NO (Wilson et al., 2008). Several factors from the parasite participate in this evasion strategy, depending on the different *Leishmania* species. *L. donovani* LPG prevents the assembly of the NADPH oxidase complex (**Figure 2B**) (Lodge et al., 2006), excludes the proton-ATPase from the phagosome (Vinet et al., 2009), and reduces its fusion with the endosome (Desjardins and Descoteaux, 1997; Scianimanico et al., 1999), what has also been demonstrated for *L. major* LPG (Dermine et al., 2000). Moreover, *L. donovani* LPG promotes the accumulation of periphagosomal F-actin (Holm et al., 2001) (Winberg et al., 2009), avoiding in this manner the phagosome acidification and favoring the parasite intracellular survival (**Figure 2B**). The *L. donovani* metalloprotease GP63 (a surface and secreted glycoprotein of 63 kDa) downregulates miR-494, which induces Rab5a expression in the phagosome to prevent lysosome fusion (**Figure 2C**) (Verma et al., 2017). Moreover, the cysteine peptidase B from *L. mexicana* regulates GP63 expression, thus indirectly influencing phagosome formation (Casgrain et al., 2016). *Leishmania* can also exploit host sphingolipids and lipid droplets as energy source and to neutralize the acidic environment inside the phagolysosome (Ali et al., 2012; Rabhi et al., 2016).

To proliferate intracellularly, *Leishmania* amastigotes require iron and arginine-derived polyamines, essential nutrients for their survival within the macrophage phagolysosome (Iniesta et al., 2001; Huynh and Andrews, 2008). *L.* amazonensis compensates the host iron efflux pump by the activation of its own iron transporters, LIT1 and LIT2, providing their mitochondria with iron to generate ROS and regulate the differentiation of virulent *Leishmania* amastigotes (**Figure 2D**) (Huynh et al., 2006; Mittra et al., 2013; Mittra et al., 2016; Mittra et al., 2017). During infection, the macrophage arginine pool is utilized to produce metabolites (NO and polyamines) for the host defense and its suppression, respectively. *Leishmania* infection up-regulates arginase activity in host macrophages, which favors polyamine synthesis and subverts NO synthase-dependent killing by competing for arginine (Gaur et al., 2007; Reguera et al., 2009; Rogers et al., 2009; Badirzadeh et al., 2017). In fact, the arginase encoded by the parasite can influence macrophage responses (Boitz et al., 2017). Polyamines contribute to proliferation and to the synthesis of anti-oxidants, such as trypanothione, which neutralize ROS and enable *L. donovani* amastigote survival inside macrophage phagolysosomes (**Figure 2E**) (Colotti and Ilari, 2011; Goldman-Pinkovich et al., 2016). However, this mechanism seems to be not essential for the survival of *L. donovani* amastigotes (Boitz et al., 2017), but it is relevant during the promastigote stage.

### Modulating Macrophage Microbicide Response

Once inside the macrophage, *Leishmania* modulates the pattern of cytokine secretion and inhibits the generation of NO and ROS to increase its survival inside the host. *L. major* promastigotes inhibit IL-12 while promoting IL-10 and TGF-β production from infected host macrophages (**Figure 2F**) (Reiner et al., 1994; Carrera et al., 1996). Most of these immunosuppressive actions depend on *Leishmania*-mediated host protein tyrosine phosphatases (PTPs) or phosphatidyl inositol-3 kinase (PI3K) recruitment, which leads to inhibition of JAK/STAT or MAPK signaling pathways, thus transforming macrophages to anti-inflammatory state as described for *L. donovani* and *L. amazonensis* (Nandan and Reiner, 1995; Blanchette et al., 1999; Nandan et al., 1999; Forget et al., 2006; Ruhland and Kima, 2009; Calegari-Silva et al., 2015).

Toll-like receptors (TLRs) expressed on innate immune cells are critical for *Leishmania* recognition, which determines the outcome of the infection (Faria et al., 2012). As a strategy to modulate the TLR response, depending on the species, *Leishmania* recruits suppressors of the cytokine signaling family proteins, SOCS-1 and SOCS-3, activates host de-ubiquitinating enzyme A20 or the Src homology 2 domain phosphotyrosine phosphatase 1 (SHP-1) (**Figure 2F**) (de Veer et al., 2003; Lapara and Kelly, 2010; Shweash et al., 2011; Srivastav et al., 2012, 2015). Moreover, *L. donovani* suppresses p38 phosphorylation while activates ERK1/2, resulting in inhibition of TLR2 and TLR4-stimulated IL-12 and increase in IL-10 production (Chandra and Naik, 2008). Similarly, the activation of the ERK and MAPK pathway in response to IgG-opsonized *L. amazonensis* boosts IL-10 production (Yang et al., 2007). Several virulence factors derived from the parasite have been implicated in this process; LPG inhibits protein kinase C (Descoteaux and Turco, 1999), and stimulates ERK (Severn et al., 1992; Proudfoot et al., 1996; Feng et al., 1999; Prive and Descoteaux, 2000; Delgado-Dominguez et al., 2010), favoring downregulation of iNOS and IL-12 production. *L. donovani* amastigotes similarly inhibit IL-12 production despite lacking LPG on their surface, indicating that other parasite-derived molecules are involved in PKC activity inhibition (Olivier et al., 1992). For example, altering macrophage intracellular ceramide homeostasis by *L. donovani* results in impaired PKC signaling (**Figure 2G**) (Ghosh et al., 2001).

Similar to other *Leishmania* factors, like the elongation factor-1alpha (EF-1alpha) and fructose-1,6-bisphosphate aldolase described in *L. donovani* (Nandan et al., 2002, 2007), *L. major* GP63 can also promote the activation and recruitment of host PTPs, like SHP-1, which suppresses several kinase pathways, inhibiting several microbicidal macrophage functions (Gomez et al., 2009) (**Figure 2F**). Moreover, GP63 can inhibit macrophages inflammatory response through mTOR signaling pathway, which regulates the IL-12/IL-10 axis (Jaramillo et al., 2011; Cheekatla et al., 2012). GP63 also mediates proteolysis of some macrophage transcriptions factors, like AP-1 and NF-κB, and adaptor molecules, such as Dok family proteins (Gregory et al., 2008; Contreras et al., 2010; Alvarez de Celis et al., 2015). Using the same mechanism, GP63 also modulates protein tyrosine kinases and PKC activity (Chawla and Vishwakarma, 2003; Halle et al., 2009). In addition, the *L. mexicana* cysteine peptidase B promotes SHP-1 function in the macrophage, inhibiting NF-κB signaling and consequently IL-12 and NO production (Cameron et al., 2004; Abu-Dayyeh et al., 2008, 2010). In addition, the glycosylphosphatidylinositol structure common for different *Leishmania* surface molecules (including LPG and GP63) inhibits TNF expression and dampens macrophage response to infection (Tachado et al., 1997). The kinetoplastid membrane protein-11 is another pathogenicity factor expressed in different *Leishmania* spp., including *L. amazonensis* amastigote stage (Matos et al., 2010) that increases IL-10 production and arginase activity while reduces NO production by macrophages (Lacerda et al., 2012). At this stage, *L. amazonensis* amastigotes can expose phosphatidylserine analogs, promoting TGF-β and IL-10 and inhibiting NO synthesis (**Figure 2H**) (Wanderley et al., 2006).

*Leishmania* can also target macrophage membrane-bound receptors to subvert the inflammatory response. Sialic acids in the parasite surface bind to Siglecs receptors on macrophages to dampen the immune response. Sialic acids recognition by Siglec-5 reduces levels of ROS, NO generation and promotes a Th2-prone cytokine response to *L. donovani* (**Figure 2I**) (Roy and Mandal, 2016). The C-type lectin receptor (CLR) SIGNR3 is targeted by *L. infantum* to inhibit Dectin-1-mediated IL-1β secretion, favoring parasite survival (**Figure 2J**) (Lefevre et al., 2013). Mannose receptor (MR) expressed by dermal macrophages is targeted by a non-healing strain of *L.* major. These cells are permissive for parasite grow even in a Th1-immune environment, affecting the severity of cutaneous disease (Lee et al., 2018). In addition, the engagement of complement receptors (CRs) type 1 and type 3 by *L. major* inhibits respiratory burst and IL-12 production (Da Silva et al., 1989; Ricardo-Carter et al., 2013) (**Figure 2I**). Moreover, the engagement of the Fc receptor (FcγR) by opsonized-parasites promotes IL-10 and TGF-β production by *L. major* infected macrophages (**Figure 2K**) (Padigel and Farrell, 2005). *L. amazonensis* and *L. major* can also induce the expression of CD200 in macrophages, mediating iNOS inhibition and promoting the virulence of the parasites (Cortez et al., 2011). Additional mechanisms like secretion of exosomes or microRNA-mediated post-transcriptional regulation of inflammatory immune response genes have been described for *L. donovani* and *L. amazonensis*, which can modulate cytokine and NO generation by macrophages (Silverman et al., 2010; Muxel et al., 2017; Tiwari et al., 2017).

### Modulating Macrophage Antigen Presentation and Costimulatory Signals

Another way used by *Leishmania* to perpetuate its presence inside the host is suppressing T cell-mediated immune responses by inhibiting presentation of *Leishmania* antigens in major histocompatibility complex (MHC) and dampening costimulatory signals provided by macrophages. Infection of macrophages affects their membrane lipid rafts fluidity and the disposition of MHC class II (MHC-II) molecules, leading to defective antigen presentation and T cell priming (Courret et al., 1999; Chakraborty et al., 2005; Roy et al., 2016). In addition, cysteine proteases from *L. amazonensis* and *L. donovani* amastigotes contribute to this process by degrading MHC-II molecules (**Figure 2L**) (De Souza Leao et al., 1995; Antoine et al., 1999). *L. donovani*-infected macrophages exhibit decreased expression of the co-stimulatory molecule B7-1 (Saha et al., 1995).

### Increasing Macrophage Survival

*Leishmania* has evolved several mechanisms to extend the survival of the infected macrophages. Programmed death-1 receptor (PD-1), which mediates T-cell exhaustion, is negatively modulated by *L. donovani* to avoid macrophage apoptosis (Roy et al., 2017). In addition, *L. donovani* triggers AKT activation of the anti-apoptotic β-catenin, inhibiting the pro-apoptotic transcriptional regulator FOXO-1 (Gupta et al., 2016). *L. donovani* also prevents mitochondria-dependent apoptosis by inducing anti-apoptotic protein MCL-1 (Giri et al., 2016). Some factors encoded by the parasite, like the orthologs of the cytokine macrophage migration inhibitory factor (MIF), are involved in blocking macrophage apoptosis and prevent clearance of internalized parasites upon *L. major* infection (Holowka et al., 2016).

## Dendritic Cells Commanding Immunity Against *Leishmania*

Dendritic cells play a unique role in the immune system as antigen presenting cells that promote and sustain adaptive immunity while contribute at the same time to the induction of tolerance to self-antigens. DCs uptake and process *Leishmania* parasites or their antigens and subsequently migrate to lymph nodes (LNs) to prime T cells. *Leishmania* sensing by DCs triggers IL-12p70 production in both human and mouse DCs, a key cytokine to prime and maintain Th1 responses that ultimately lead to the control of the parasite (Gorak et al., 1998; von Stebut et al., 1998; Marovich et al., 2000; Leon et al., 2007). In order to escape, *Leishmania* parasites target DC activation either being silent or even inhibiting DC activation, motility and migration to draining LNs (Ponte-Sucre et al., 2001; Jebbari et al., 2002; De Trez et al., 2004; Revest et al., 2008; Sanabria et al., 2008; Figueiredo et al., 2012; Hermida et al., 2014; Iborra et al., 2016; von Stebut, 2017). DC-*Leishmania* interaction can vary depending on the different DC subset involved, as they are equipped with different pattern recognition receptors. In addition, several *Leishmania* species and different strains might be endowed with different pathogen associated molecular patterns and/or immune evasion strategies. Moreover, the interaction of the parasite and DCs can be direct or indirect, through other infected cells, and even the sandfly saliva may also modulate DCs function.

**FIGURE 3:**
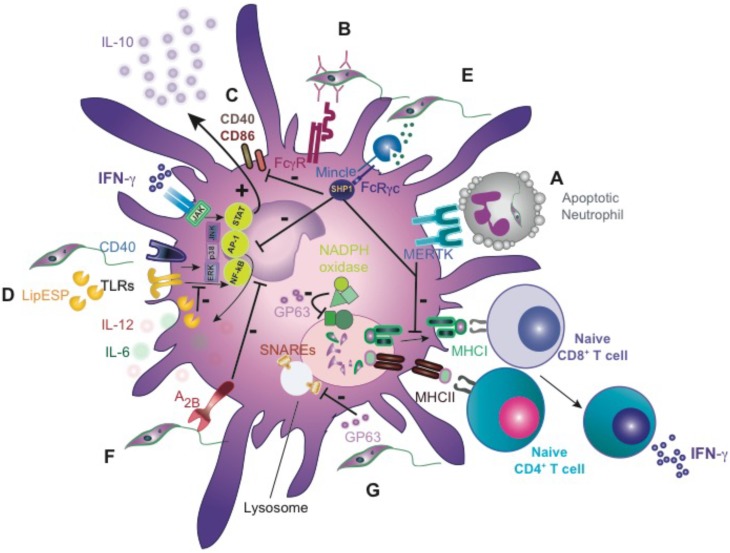
*Leishmania* inhibits DC function. **(A)** Apoptotic cell clearance of *L. major*-infected neutrophils by DCs shuts down cross-priming of CD8 T cells *in vitro* by MERTK-dependent signaling. **(B)** Internalization of opsonized *L. major* by DCs via FcγR promotes DC activation and IL-12 production. **(C)** Downregulation of costimulatory molecules, CD40 and CD86 upon *L. major* infection *in vitro*. **(D)** *L. infantum excreted*/secreted factors (LipESP) reduce LPS- and CD40-mediated responses. **(E)** Binding of soluble *Leishmania* ligand(s) to Mincle promotes SHP-1 interaction and inhibitory signaling. **(F)** *L. amazonensis* exploits A(2B) receptor to inhibit DCs function. **(G)** GP63 cleaves SNAREs protein, preventing the assembly of the NADPH oxidase complex. FcγR, Fcγ receptor; MERTK, tyrosine-protein kinase Mer; LipESP, *L. infantum* excreted/secreted proteins; A(2B) receptor, adenosine; GP63, glycoprotein 63; SNAREs, soluble NSF attachment protein receptor.

*L. major* inoculation induces a huge infiltration of neutrophils that phagocytose the majority of parasites but fails to kill them, although this is not the case for other *Leishmania* species (Regli et al., 2017). DCs reaching the inflammation site would thus mainly encounter apoptotic neutrophils harboring intracellular parasites. The capture of infected neutrophils by DCs in the skin acts as a key mechanism to inhibit their functions, delaying the development of adaptive immunity (**Figures 1I**, **3A**) (Ribeiro-Gomes et al., 2012). In fact, treatment of mice with two neutrophil-depleting antibodies, the GR-1-specific antibody RB6-8C5, which recognizes an epitope shared by Ly6G and Ly6C, and the Ly6G-specific antibody, 1A8, just before infection augments DCs maturation in the skin and the priming of *L. major* specific CD4^+^ T cells *in vivo*, which correlates with faster parasite clearance (Peters et al., 2008; Ribeiro-Gomes et al., 2012). Moreover, uptake of infected neutrophils inhibits DC maturation and their subsequent function as cross-priming DC *in vivo* (Ribeiro-Gomes et al., 2015). Upon *L. major* infection (Friedlin strain FV1), the engagement of the receptor tyrosine kinase Mer (MERTK) on the DCs phagocytosing apoptotic neutrophils led to the impaired capacity for CD8^+^ T-cell priming *in vitro*. MERTK acted as a tolerogenic receptor in resting macrophages and in the absence of inflammation (**Figures 1I**, **3A**) (Zagorska et al., 2014). Interestingly, the related protozoan parasite *Toxoplasma gondii* does not elicit this inhibitory response to the same extent (Ribeiro-Gomes et al., 2015). In addition, the parasites co-evolved a strategy where the virulent inoculum comprises viable and dying promastigotes, which expose phospholipids analogs to phosphatidylserine (Weingartner et al., 2012), a prototypical apoptotic eat-me signal promoting phagocytosis in a “silent” way. Thus, DCs can also engulf free extracellular *Leishmania* promastigotes (Ng et al., 2008).

### *Leishmania* Modulates DC Maturation and Migration

Upon recognition of pathogen-derived molecules, DCs migrate to lymphoid tissues and undergo a process of “maturation” that enhances antigen processing and presentation, expression of costimulatory molecules and cytokine secretion, governing the fate of adaptive immunity. Several *in vivo* studies demonstrate the importance of fully activated migratory DCs (CD86^high^, CD40^high^, CCR7^+^, and IL-12^+^) in activation of NK cells and in the generation of protective Th1 responses against *Leishmania* parasites (Soong, 2008). Therefore, incomplete and delayed DC maturation could favor the establishment and amplification of *Leishmania* infection before the onset of immune responses. Of note, in an experimental model mimicking natural infection (low number *L. major* metacyclic promastigotes challenged in the ear dermis) a silent phase was observed with parasite replication in the absence of an inflammatory response. In this model, IL-12^+^ DCs were not detected until week 4 post-infection, peaking at week 6 and preceding full development of T cell-associated IFN-γ release (Belkaid et al., 2000). *L. major* internalization by DCs is facilitated by IgG via FcγRI and FcγRIII, and engagement of these receptors is required for development of Th1 dependent immunity (**Figure 3B**) (Woelbing et al., 2006). However, it is unknown when B-cell priming against *Leishmania* occurs, and whether natural IgGs can opsonize *Leishmania* and promote DC engulfment. The existence of a “silent phase” suggests that *Leishmania* is able to modulate DC maturation, motility or migration. In fact, upon *in vitro* infection with high doses of *L. major* promastigotes, DCs did not exhibit upregulation of MHC class I/II, costimulatory molecules, such as CD40, CD86, as well as release of proinflammatory cytokines (**Figure 3C**) (von Stebut et al., 1998). Similarly, the presence of live *L. amazonensis* parasites during human DC differentiation *in vitro* decreased CD80 expression and IL-6 secretion (Favali et al., 2007). Mouse bone marrow-derived DCs (BMDCs) infected with *L. amazonensis*, *L. braziliensis*, *L. major*, or *L. infantum* metacyclic promastigotes showed decreased MHC-II and CD86 expression, and exhibited an impaired ability to induce T-cell proliferation (Neves et al., 2010; Figueiredo et al., 2012). In addition, some *L. infantum* excreted/secreted proteins (LipESP) reduced the ability of human DCs to respond *in vitro* to LPS, inhibiting maturation and IL-12p70 production (**Figure 3D**) (Markikou-Ouni et al., 2015). Exosomes from *L. donovani* failed to prime monocyte-derived human DCs to drive the differentiation of naive CD4 T cells into IFN-γ-producing Th1 cells *in vitro*. Interestingly, vesicles from *L. donovani* deficient in HSP100, which exhibit a distinct protein cargo, have more proinflammatory phenotype in human DCs *in vitro* (Silverman et al., 2010).

The outcome of the DC interaction with the parasite depends on the *Leishmania* species and the developmental stage. DC maturation is not observed upon *L. major* promastigote infection, but can be induced by *L. major* amastigotes *in vitro* (von Stebut et al., 1998). In contrast, DCs infected with *L. mexicana* amastigotes do not show detectable levels of IL-12, or any other signs of activation (Bennett et al., 2001). Likewise, *L. amazonensis* amastigotes failed to induce CD40-dependent IL-12 *in vitro* production in DCs (**Figure 3D**) (Qi et al., 2001; Boggiatto et al., 2009). Notably, DCs infected with *L. amazonensis* promastigotes displayed a “semi-activation” phenotype, produced relatively low levels of IL-12, and preferentially induced pathogenic CD4^+^ T cells (Xin et al., 2007). *L. amazonensis* amastigote-infected DCs were less mature and with lower antigen presenting capacity *in vitro* compared with promastigote-infected DCs (Xin et al., 2008). In contrast to parasite extract stimulation or infection, internalization of antibody-opsonized *L. amazonensis* promastigotes or amastigotes induces DC maturation, as shown by the over-expression of costimulatory, adhesion and MHC-II (Prina et al., 2004).

The mechanisms that *Leishmania* uses to sabotage DCs are still not fully defined. *L. mexicana* infection of the DC line DC2.4, inhibits the MAPK-signaling cascade, decreasing antigen-presentation capacity and IL-12 secretion (**Figure 3D**) (Contreras et al., 2014). This inhibition is mediated by the activation of PTPs. *Leishmania* can be detected by different PRRs, such as TLRs, CLRs and opsonizing antibodies via Fc receptors, which trigger activating and/or inhibitory signals (Woelbing et al., 2006; Lefevre et al., 2013). Mincle (Clec4e) mediates dampening of DC activation and migration following sensing of a ligand released by *Leishmania* (Iborra et al., 2016). Mincle couples to the Fc receptor γ (FcRγ) chain that bears immunoreceptor tyrosine-based activation motif (ITAM). Upon canonical signaling through Mincle, tyrosine residues in the FcRγ chain are phosphorylated by Src-family kinases, followed by the recruitment and activation of the kinase Syk, which generates an activating signal that boosts inflammation (Sancho and Reis e Sousa, 2012, 2013). Notably, upon recognition of *Leishmania* ligand, Mincle shifts to an inhibitory ITAM configuration that recruits SHP-1 and dampens DC activation and migration induced by heterologous receptors sensing activating signals from *Leishmania* (**Figure 3E**) (Iborra et al., 2016). Thus, we observed a more robust IFN-γ-producing-CD4^+^ T cell response, milder dermal pathology and 10-fold reduction of the parasite burden compared to wild-type mice. Selective loss of SHP1 in CD11c^+^ cells phenocopies enhanced adaptive immunity to *Leishmania* (Iborra et al., 2016). Another way to dampen DC activation related to purinergic signaling has been demonstrated in DCs infected with *L. amazonensis*. Whereas extracellular ATP induces inflammation, adenosine is an important anti-inflammatory mediator. In the presence of MRS1754, a highly selective A(2B) adenosine receptor antagonist, DCs exhibit an increased expression of MHC-II, CD86 and CD40, enhancing their ability to induce T-cell proliferation. In conclusion, A(2B) receptor activation may be used by *Leishmania* to inhibit DC function and evade the immune response (**Figure 3F**) (Figueiredo et al., 2012).

*Leishmania* may also subvert adaptive immunity by interfering with DC migration. In the steady state, dermal DCs are highly motile, continuously crawling through the interstitial space. Intradermal delivery of *L. major* immobilizes dermal DCs (Ng et al., 2008). Products secreted by *L. major* promastigotes inhibit the motility of DCs by up to 93%, in a dose-dependent and reversible manner (Jebbari et al., 2002). Co-incubation with *Leishmania in vitro*, changes the migratory pattern of DCs when they are adoptively transferred to mice (Hermida et al., 2014). Similarly, we described that *Leishmania* inhibits DCs migration via Mincle (Iborra et al., 2016). DCs from mice with chronic *L. donovani* infection fail to migrate from the marginal zone to the periarteriolar region of the spleen. However, DCs eventually migrate and promote Th1 cell immunity and macrophage microbicidal activity (Leon et al., 2007).

Different DC subsets can coordinate the mounting of anti-*Leishmania* response. *L. major* infection recruits monocytes to the dermis that generate Th1-promoting dermal monocyte-derived DCs (Leon et al., 2007). In addition, cDC1s (Batf3-dependent DCs) are essential for the control of *L. major* (Ashok et al., 2014; Martinez-Lopez et al., 2015). Although this DC subset does not seem essential for Th1 or CTL priming (Martinez-Lopez et al., 2015), probably because cDC1 are resistant to infection (Henri et al., 2002), they excel in IL-12 production, which is crucial for maintenance of local Th1 immunity against *L. major* infection (Martinez-Lopez et al., 2015).

### Interfering With CD8^+^ T Cell Cross-Priming

CD8^+^ T lymphocytes are components of the adaptive immune response that play an important role in protection against intracellular pathogens. The role of CD8^+^ T cells in the primary control of *Leishmania* is still controversial, given the different results obtained in different infection models. CD8^+^ T cells contribute to parasite control in visceral leishmaniasis (Stäger and Rafati, 2012), probably by recruiting inflammatory cells and maintaining granulomas. CD8^+^ T cells also contribute to parasite clearance against low doses of *L. major* (Belkaid et al., 2002b), where they also contribute to the cutaneous pathology associated to the infection, and even exacerbate disease (Novais and Scott, 2015). However, *L. donovani* induces defective antigen-specific CD8^+^ T cell responses, with a very limited clonal expansion (Joshi et al., 2009), compared with viral infections or following injection of irradiated *Plasmodium* (Sano et al., 2001). In fact, visceral leishmaniasis patients do not show CD8^+^ T cell effector responses (Gautam et al., 2014).

Limited and poor *Leishmania* antigen-processing and presentation into MHC Class I, could be one potential explanation. Processing of *Leishmania* antigens occurs in a TAP-independent, intraphagosomal pathway that is less efficient and requires higher amounts of secreted antigen than the endoplasmic reticulum-based, TAP-dependent cross-presentation pathway (Bertholet et al., 2006). In addition to other mechanisms discussed above, the major surface metalloprotease of *Leishmania* GP63 cleaves a subset of SNAREs, including VAMP8. The inactivation of VAMP8 prevents the assembly of the NADPH oxidase complex (NOX2), which is critical to limit the acidification in these cross-presentation compartments (**Figure 3G**) (Matheoud et al., 2013; Matte et al., 2016). The inhibition of acidification is critical to prevent the complete and premature destruction of MHC class I epitopes by the protease activity (Savina et al., 2006). As a consequence, the cross-presentation of *Leishmania* antigens on MHC class I molecules is actively inhibited by the parasite. CD8^+^ T cells undergo a second round of activation, become dysfunctional, and ultimately die from exhaustion during infection (Joshi et al., 2009). Given that high and constant antigenic stimulation causes CD8^+^ T cell “exhaustion” during chronic viral infections (Mueller and Ahmed, 2009), we could speculate that *Leishmania* antigens might be available for cross-presentation from other sources, like death infected macrophages.

## Fighting Back Immune Evasion by Vaccination

Inoculation of live virulent *L. major* parasites causing auto-curing cutaneous leishmaniasis lesions, a procedure known as leishmanization (Khamesipour et al., 2005) is the only efficient vaccine that induces immunity in human subjects (Saljoughian et al., 2014; Mendonca, 2016). Resistance to reinfection with *L. major* in mice has been linked to the induction of parasite persistence by CD4^+^CD25^+^ regulatory T cells secreting IL-10 (Belkaid et al., 2001, 2002a). The presence of small number of parasites in macrophages and DCs after primary challenge (Mandell and Beverley, 2017) preserves the concomitant immunity necessary to induce long-lasting defense (Sacks, 2014), consisting of migrating IFN-γ-producing effector T cells to the site of reinfection (Uzonna et al., 2001; Peters et al., 2014; Romano et al., 2015), and CD4^+^ resident memory T cells in the infected skin (Glennie et al., 2015) that can further recruit effector T cells and inflammatory monocytes to the infected dermal site (Glennie et al., 2017).

Generation of long-lasting cellular immunity is the main objective of vaccines based on parasite proteins or extracts. Immunotherapy using DC-based vaccination is an emerging potent approach for harnessing the potential of a patient’s own immune system to induce protection. DCs can be pulsed with parasite extracts alone (Ahuja et al., 1999; Carrion et al., 2008; Majumder et al., 2012; Masic et al., 2012), combined with adjuvants such as CpG-ODN (Carrion et al., 2008; Agallou et al., 2011, 2012; Majumder et al., 2012; Masic et al., 2012) or peptidoglycan (ligand of the TLR-2) (Jawed et al., 2016) or in DCs engineered to secrete IL-12 (Ahuja et al., 1999). These different treatments boost their immunogenicity in murine models (Ahuja et al., 1999; Majumder et al., 2012; Jawed et al., 2016), dampening IL-10 responses associated to parasite infection (Schwarz et al., 2013), and decreasing the tissue damage induced by the inflammatory response after infective challenge in vaccinated animals (Masic et al., 2012). Due to the high cost of these procedures, an alternative to the use of DCs primed with recombinant parasite proteins in humans will be to target *Leishmania* proteins to DCs by constructing recombinant chimeras, such as recombinant antibodies recognizing DC-specific receptors and containing leishmanial proteins. Using this strategy, antigen-specific CD4^+^ T cells producing IFN-γ, IL-2, and TNF were found in vaccinated mice (Matos et al., 2013).

## Concluding Remarks

Myeloid cells, including neutrophils, monocytes, macrophages and DCs, orchestrate the generation of protective innate and adaptive immunity against *Leishmania*. Neutrophils are the first line of defense and generate an inflammatory response that restrains the parasite but, at the same time, and for some *Leishmania* species, neutrophils may act as carriers that facilitate silent infection of macrophages (Laskay et al., 2003, 2008; John and Hunter, 2008). Once within the macrophage, and depending on the *Leishmania* species, parasites delay phagosome formation and maturation, preventing phagosome acidification and action of proteases, while securing the nutrients needed for their survival. Moreover, the parasites modulate the pattern of cytokine secretion and inhibit the generation of NO and ROS, while extending the survival of the infected macrophages. Similarly, *L. major*-infected neutrophils are silently phagocytozed by DCs in the skin and inhibit DC maturation and migration, delaying the development of adaptive immunity (Ribeiro-Gomes et al., 2012, 2015; Peters et al., 2014). Both monocyte-derived DCs and cDC1s are essential for the generation of Th1 immunity resulting in the control of *L. major* (Leon et al., 2007; Ashok et al., 2014; Martinez-Lopez et al., 2015). *Leishmania* acts at different levels to inhibit DCs, including dampening the MAPK pathway, decreasing antigen presentation capacity, IL-12 secretion and migration, being this inhibition mediated by the activation of PTPs (Contreras et al., 2014; Iborra et al., 2016). Understanding which DC populations are key to trigger and achieve immunity to *Leishmania* and how parasites inhibit their activation and migration will help to improve a rational design of vaccines aimed to counteract parasite virulence factors, along with the use of the most adequate adjuvants.

## Author Contributions

MM-L, MS, SI, and DS conceived and wrote the manuscript. MM-L did the figures.

## Conflict of Interest Statement

The authors declare that the research was conducted in the absence of any commercial or financial relationships that could be construed as a potential conflict of interest.
